# Supplementary material for: Effects of Dietary Rare Earth Chitosan Chelate on Performance, Egg Quality, Immune and Antioxidant Capacity, and Intestinal Digestive Enzyme Activity of Laying Hens
Source: Polymers (Basel). 2023 Mar 23;15(7):1600. doi: 10.3390/polym15071600 (PMC10097366; doi:10.3390/polym15071600)
Supplement: Supplementary file 1 [file polymers-15-01600-s001.zip › polymers-2251160-supplementary.pdf]

Supplemental Table S1. The list of commercial assay kits purchased.

Index	Name of kits	Trademark	Cat No.
GSH-PX	Glutathione peroxidase (GSH-PX) assay kit (colorimetry)	Nanjing Jiancheng	A005
T-AOC	Total antioxidant capacity (T-AOC) assay kit (ABTS)	Nanjing Jiancheng	A015-2-1
SOD	Total superoxide dismutase (SOD) assay kit (WST-1)	Nanjing Jiancheng	A001-3-2
MDA	Malondialdehyde (MDA) assay kit (TBA)	Nanjing Jiancheng	A003-1-2
GSH	Reduced glutathione (GSH) assay kit (spectrophotometry)	Nanjing Jiancheng	A006-1
AMS	α- Amylase (AMS) assay kit (starch-iodine colorimetry)	Nanjing Jiancheng	C016-1-1
Trypsin	Trypsin assay kit (UV colorimetry)	Nanjing Jiancheng	A080-2-2
LPS	Lipase (LPS) assay kit (colorimetry)	Nanjing Jiancheng	A054-1-1
sIgA	Chicken secretory immunoglobulin A (sIgA) ELISA kit	MLBio	ml023618
IL-2	Chicken interleukin-2 (IL-2) ELISA kit	MLBio	ml042736
IL-6	Chicken interleukin-6 (IL-6) ELISA kit	MLBio	ml042757
TFT-α	Chicken tumor necrosis factor α (TNF- α) ELISA kit	MLBio	ml002790
IgA	Chicken immunoglobulin A (IgA) ELISA kit	MLBio	ml002792
IgM	Chicken immunoglobulin M (IgM) ELISA kit	MLBio	ml002781
IgG	Chicken immunoglobulin G (IgG) ELISA kit	MLBio	ml042771
